# Pathological and Incidental Findings in 403 Taiwanese Girls With Central Precocious Puberty at Initial Diagnosis

**DOI:** 10.3389/fendo.2020.00256

**Published:** 2020-05-05

**Authors:** Chiao-Fan Chiu, Chao-Jan Wang, Yi-Pin Chen, Fu-Sung Lo

**Affiliations:** ^1^Department of Pediatrics, Chang Gung Memorial Hospital, Taoyuan, Taiwan; ^2^Graduate Institute of Clinical Medicine Sciences, College of Medicine, Chang Gung University, Taoyuan City, Taiwan; ^3^College of Medicine, Chang Gung University, Taoyuan City, Taiwan; ^4^Department of Medical Imaging and Intervention, Chang Gung Memorial Hospital, Taoyuan, Taiwan; ^5^Department of Obstetrics and Gynecology, Keelung Chang Gung Memorial Hospital, Keelung, Taiwan

**Keywords:** Taiwan, prevalence, brain MRI, girl, central precocious puberty (CPP)

## Abstract

**Background:** Whether girls with central precocious puberty (CPP) should undergo routine brain magnetic resonance imaging (MRI) to identify potential intracranial pathologies is controversial.

**Aims:** To evaluate the brain MRI results of girls with CPP and identify the clinical and endocrine predictors of brain abnormalities.

**Methods:** This retrospective study obtained data from pediatric endocrine clinics at Chang Gung Children's Hospital. From 1997 and 2017, 403 girls were consecutively diagnosed with CPP. After the exclusion of patients with a history of central nervous system (CNS) insults or associated neuropsychiatric symptom or signs, we studied the prevalence of brain abnormalities in 251 girls with CPP who received detailed MRI examinations of the hypothalamus and pituitary area. We also recorded the demographic data of the participants, including the onset of puberty; initial pubertal status; height; weight; uterus and ovary sizes; and basal luteinizing hormone (LH), follicle-stimulating hormone (FSH), estradiol levels, and the response to GnRH stimulation test.

**Results:** Among the 251 girls with CPP, we observed no brain alterations in 190 (75.70%), abnormalities in the hypothalamic-pituitary (H-P) area in 54 (21.51%), and abnormalities in the non-H-P area in 7 (2.79%). Among the 54 patients that had H-P lesions, we noted pathological findings related to CPP (hypothalamic hamartoma) in only one (0.4%) girl aged below 6 years old. We also identified incidental findings in the other girls with H-P lesions, including non-functioning pituitary microadenomas (12.35%), pituitary pars intermedia cysts (4.38%), Rathke's pouch cysts (1.99%), pituitary hypoplasia (1.59%), and pineal gland cysts (0.8%). The patients that had non-H-P lesions were found to have arachnoid cysts (1.59%), Chiari I malformation (0.4%), prepontine nodule (0.4%), and choroidal fissure cyst (0.4%). Of all the patients with brain lesions, 45 (73.77%) underwent regular MRI follow-up. While none of the H-P and non-H-P lesions showed progression, 19.67% of these regressed during the follow-up. None of the participants exhibited other hormonal abnormalities or underwent surgery.

**Conclusion:** The prevalence of true pathological brain lesions related to CPP in girls without prior symptoms or signs of CNS lesions was low (0.4%). None of the girls with intracranial lesions required further intervention besides the GnRH agonist treatment. These data question the routine use of brain MRI in all girls with CPP, especially in those who are healthy without neurologic symptoms.

## Introduction

Central precocious puberty (CPP), which affects 1 in 5,000–10,000 girls, is caused by the premature activation of the hypothalamic-pituitary-gonadal axis (1, 2). Girls with CPP typically experience the onset of secondary sexual characteristics before the age of 8 years (1). Given that central nervous system (CNS) abnormalities are much more likely (40–75%) to accompany CPP diagnoses in boys (1, 3–5), CNS imaging is usually conducted for all boys with CPP. However, brain MRI may not necessary for all girls with CPP due to a lower risk of having other CNS abnormalities. In ~90% of girls, CPP is idiopathic (1). The prevalence of unsuspected intracranial lesions is 8–13% in girls and decreases with age (6). Among girls with CPP (onset age of 6–8 years), unsuspected pathology and tumors were only involved in 2–7% and 1% of the them, respectively (4). However, brain MRI continues to be routinely performed to identify potential intracranial lesions in all girls with CPP (6–9).

This study evaluated the prevalence and type of intracranial findings in girls with CPP with no previous CNS lesions, or related symptom and signs, and examined the necessity of routine brain MRI in girls with CPP.

## Patients and Methods

This retrospective study was approved by the Institutional Review Board of Chang Gung Memorial Hospital (Taiwan); at that hospital, the enrollees attended pediatric endocrine clinics between 1997 and 2017. During that period, a total of 403 girls were consecutively diagnosed with CPP, 251 of whom underwent detailed brain MRI studies of the hypothalamus and pituitary area. On the basis of the MRI results, we studied the prevalence of brain abnormalities in these patients. Patients with previous CNS insults, associated neuropsychiatric symptom or signs, and an absence of MRI data were excluded from this study.

The diagnosis of CPP was made based on a clinical evaluation, which included the detailed history taking of the patient and their caregivers followed by a physical examination and Tanner staging by pediatric endocrinologists. The recorded data included that on the onset and progression of puberty, breast development (unilateral or bilateral), pubic hair growth, and menarche. Other associated information, such as that on height velocity, sex steroid exposure, suggestive symptoms, and signs of a CNS lesion (such as headache, emesis, seizure, visual filed defect), birth history (gestational age, birth weight, birth insults), and chronic diseases was also obtained. Information regarding the onset of puberty in parents and siblings (e.g., age at menarche, growth spurt) of the study subject was also gathered.

Pubertal status was staged according to Tanner's criteria (10, 11). Height and BMI values were expressed as standard deviation scores (SDSs) with reference to the WHO standards.

We analyzed the left hand and wrist X-ray results (bone age assessment), pelvic ultrasound (uterus, ovaries, and adrenal gland assessment), serum basal LH, FSH, and estradiol levels, and the GnRH stimulation responses of the patients.

Bone age was estimated by pediatric endocrinologists using the Greulich and Pyle Atlas (12). Serum free T4, TSH, prolactin, FSH, LH, and estradiol levels were measured using commercial kits for radioimmunoassay (RIA) or the Immunelite 2000 XPi. GnRH test was performed in girls with rapid pubertal progression or advanced bone age above 2 S.D. or elevated basal estradiol level or ultrasound evidence of pubertal development of uterus and ovaries. The GnRH stimulation test was performed after the intravenous administration of 0.1 mg of Gonadorelin acetate (LH-RH Injection, TANABE). LH and FSH levels were then measured at 0, 30, 60, 90, and 120 min after injection (9, 13). An LH peak exceeding 5 mIU/mL or an LH/FSH ratio >0.66 were considered to indicate a pubertal response. A basal LH level of >0.3 mIU/mL was also considered to be an indicator of CPP (14, 15).

To identify any hypothalamic or pituitary lesions, 250 girls diagnosed with CPP underwent brain scans using gadolinium-enhanced T1- and T2-weighted MRI. Ninety-four girls underwent Brain CT and 28 girls missed image study and were excluded from this analysis.

### Statistical Analyses

Statistical analyses were conducted using SPSS version 20.0. A chi-square test and Fisher's exact test were used to conduct comparisons between the age groups. A *p* < 0.05 was considered statistically significant.

## Results

**Figure 1:**
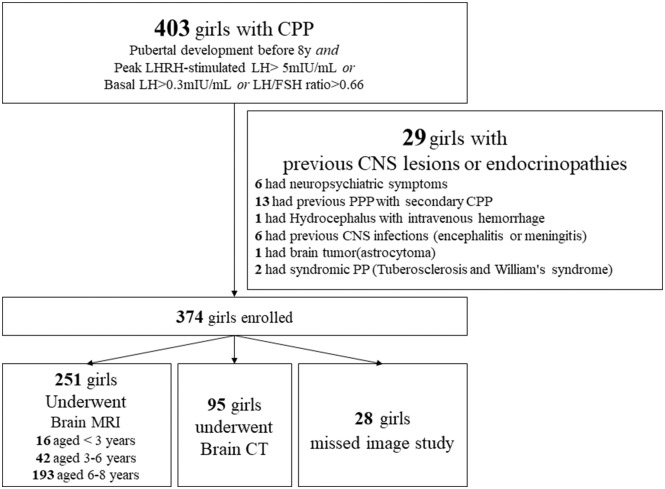
Flow diagram for the diagnosis of 403 Taiwanese girls with CPP using brain magnetic resonance imaging (MRI) or computed tomography (CT) scans. PPP, peripheral precocious puberty.

A total of 403 girls were diagnosed as having CPP, of which 29 had previous brain insults (e.g., brain tumors, CNS infection, hydrocephalus), peripheral precocious puberty (PPP; e.g., congenital adrenal hyperplasia, McCune Albright syndrome, functional ovarian cysts, ovarian teratoma) with secondary CPP, syndromic precocious puberty (tuberous sclerosis and Williams syndrome), and neuropsychiatric symptoms (Figure 1).

The mean age at pubertal onset was 6.51 ± 1.50 years (range 0.63–8.0 years). To understand the initial presentation of the patients at their first visit to the clinic, we analyzed their pubertal development staging. At this time point, the proportions of girls found to be in Tanner stage 2, 3, 4, and 5 of breast development were 49.1, 37.2, 11.2, and 2.5%, respectively. Approximately 81.4% of the girls had bilateral breast development, 10.7% had left breast development, and 7.9% had right breast development. Finally, 5.2% of girls had pubic hair growth and 2% had menarche. Other descriptive characteristics of 403 girls with newly diagnosed CPP were listed in Supplementary Appendix.

Among the 251 girls with CPP who underwent brain MRI, the age group distributions for those of 6–8 years, 3–6, years and <3 years were 76.89% (*n* = 193), 16.73% (*n* = 42), and 6.37% (*n* = 16), respectively. On the basis of MRI findings, girls were categorized into three groups: normal with no relevant abnormalities or pituitary hyperplasia alone (group A), abnormalities of the hypothalamus-pituitary (H-P) area (group B), and abnormalities of non-hypothalamus-pituitary (non-H-P) lesions (group C).

**Figure 2:**
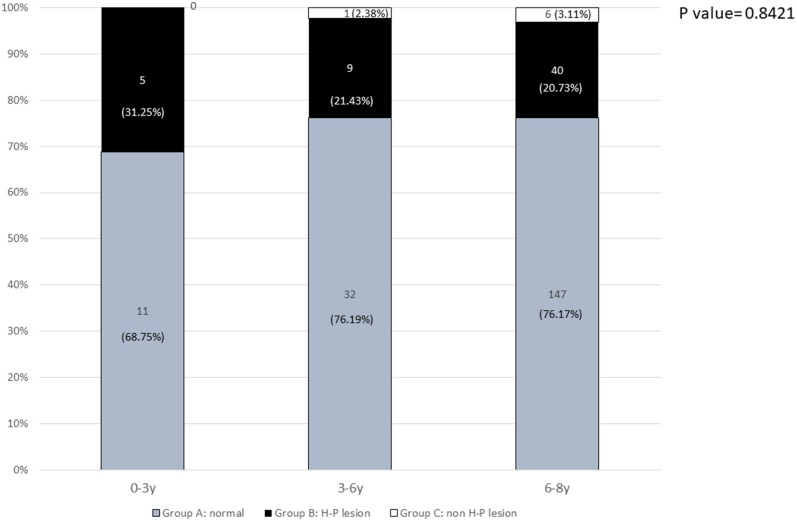
The prevalence of normal and pathological brain MRI findings in different age groups: 0–3 years, 3–6 years, and 6–8 years.

The prevalence rates of pathological findings (H-P lesions) for girls aged 6–8 years, 3–6 years, and <3 years were 20.73, 21.43, and 31.25% (*p* = 0.8421), respectively (Figure 2).

Brain MRI revealed no alterations in 190 (75.70%) of the patients. Abnormalities in the hypothalamic-pituitary area unrelated to CPP were found in 54 (21.51%) of the patients. One patient (0.4%) was identified with a hypothalamic hamartoma (HH). We revealed that 24.3% of the patients had newly diagnosed intracranial pathologies, the majority of which were incidentalomas. The reported distribution of H-P CNS alterations detected at diagnosis (except hamartoma) were as follows: pituitary microadenoma (12.35%), cyst of pituitary pars intermedia (4.38%), Rathke's pouch cyst (1.99%), pituitary hypoplasia (1.59%), and pineal gland cyst (0.8%). The patients that had non-H-P lesions were found to have arachnoid cysts (1.59%), Chiari I malformation (0.4%), prepontine nodule (0.4%), and choroidal fissure cyst (0.4%) (Table 1).

**Table 1:** Brain MRI findings for 251 Taiwanese girls with CPP.

**Group**	**Finding**	**Number (%) Total 251**	**Outcome**
A	Normal	190 (75.70%)	
	No relevant findings	156	
	Pituitary hyperplasia	34	3/33 F/U, stationary (1 → normal)
B	H-P lesions	54 (21.51%)	
	Hamartoma of the tuber cinereum	1	Loss of follow-up
	Pituitary microadenoma	31	26 F/U, stationary (10 → normal)
	Pituitary hypoplasia	4	2 F/U, stationary (1 → normal)
	Cyst of pituitary pars intermedia	11	7 F/U, stationary (1 → normal)
	Rathke's pouch cyst	5	2 F/U, stationary
	Pineal gland cyst	2	2 F/U, stationary
C	Non-H-P lesions	7 (2.79%)	
	Chiari I malformation	1	F/U, stationary
	Prepontine nodule	1	F/U, stationary
	Right choroidal fissure cyst	1	F/U, stationary
	Arachnoid cyst	4	3 F/U, stationary

*Both the initial image at diagnosis and the follow-up images are presented*.

One patient with tuber cinereum hamartoma was diagnosed with CPP at the age of 5 years and 6 months. Initial presentation included breast development that started at the age of 3 years and 6 months. Tanner stage 4 of breast development and stage 2 of pubic hair growth were determined. However, no menstruation was reported. The patient measured 132.7 cm (4.34 *SD*) in height, and the height SDS minus familial height SDS was 4.56. The patient had a significant advanced bone age of 12 years, respectively, and a basal LH and estradiol levels of 4.25 mIU/mL and 26.3 pg/mL, respectively. The peak stimulated LH level after the GnRH stimulation test was 35.2 mIU/mL and the peak stimulated LH/FSH ratio was 3.56. Apart from premature pubertal development, no other symptoms were observed before the diagnosis of tuber cinereum hamartoma. This patient was the only one found to have true organic lesions related to CPP after brain MRI study.

Most of the patients (73.77%) with incidental findings underwent MRI scans during the follow-up period. None of the girls with incidentaloma had other hormonal abnormalities, nor did they undergo surgery (Table 1).

## Discussion

In this study, we evaluated the brain MRI data of 251 Taiwanese girls with CPP. Among these 251 patients, 190 (75.70%) exhibited no alterations in the hypothalamus-pituitary area of the brain, 54 (21.51%) exhibited hypothalamus-pituitary (H-P) lesions, and 7 (2.79%) exhibited non-H-P lesions. The H-P lesions identified included pituitary microadenoma (*n* = 31, 12.35%), pituitary pars intermedia cysts (*n* = 11, 4.38%), Rathke's pouch cyst (*n* = 5, 1.99%), pituitary hypoplasia (*n* = 4, 1.59%), pineal gland cysts (*n* = 2, 0.8%), and hamartoma of the tuber cinereum (*n* = 1, 0.4%). The non-H-P lesions identified included arachnoid cyst (*n* = 4, 1.59%), Chiari I malformation (*n* = 1, 0.4%), prepontine nodule (*n* = 1, 0.4%), and choroidal fissure cyst (*n* = 1, 0.4%).

Hypothalamic hamartomas (HHs) are the commonest brain lesions that cause CPP (16). However, only one patient was found to have an HH in this study. HH is thought to induce CPP via the premature pulsatile release of ectopic GnRH (17, 18). According to a review of 36 publications detailing 124 patients with HHs, some presentations of pubertal development, such as thelarche and menarche, may precede gelastic seizures (18). Thus, CPP may be the first manifestation of HH. Currently, the standard treatment for CPP in patients with HH is GnRH agonist therapy unless surgical resection is required for intractable epilepsy. In this study, other abnormal findings in the H-P area included pituitary microadenomas (56.6%), pituitary pars intermedia cysts (20.75%), and Rathke's pouch cysts (9.43%). One girl with Angelman syndrome was reported to present with CPP and a pituitary pars intermedia cyst (as revealed by brain MRI) (19). Pituitary microadenomas were the most common brain lesions in this study (*n* = 30). Consistent with previous findings (20), the levels of gonadotropins were similar between patients with microadenomas and those without. Therefore, we considered pituitary microadenomas to be incidentalomas. In our study, luteinizing hormone(LH), follicle stimulating hormone(FSH), estradiol, prolactin level, insulin-like growth factor-1(IGF-1) level, thyroid function, ACTH and cortisol levels, as well as blood and urine osmolality were surveyed in the girls of pituitary microadenoma, none of them had other hormonal abnormalities, nor did they undergo surgery (Table 1). Therefore, we defined these microadenomas as non-functioning incidentalomas. A previous retrospective cross-sectional study of 3,528 girls reported a low prevalence (8.2%) of incidental lesions in girls with CPP. Similar to our study group, none of the identified lesions required treatment (21). According to autopsy studies, the prevalence of silent pituitary adenomas (predominantly prolactinomas) is 10.7% (range 14.7–37%). A previous study of MRI data identified pituitary “hypointensities” in 10–40% of healthy adult volunteers (22). Another study on children aged 2–16 years noted “certain” and “possible” microadenomas in 28.6 and 21.4%, respectively, of the children without hormonal abnormalities (*n* = 528) (23). In another study, 41 children aged below 18 years were identified with pituitary “incidentalomas” during evaluation of CNS symptoms and signs such as headache, of which 6 (14.6%) were microadenomas (24).

Recently, a study reported that asymptomatic non-functional pituitary lesions, including cysts, microadenomas, or possible microadenomas follow a benign clinical course in children. The authors concluded that in the absence of new endocrine or visual symptoms, repeated MRI is either not necessary or should be conducted once within the first year (22). When possible, hyperprolactinemia-inducing medications should be discontinued before imaging (22). According to the Endocrine Society guidelines (25), all patients with radiologically diagnosed pituitary adenomas should receive evaluation of the endocrine function. A formal visual field examination should be given to all patients with lesions abutting the optic nerve and chiasm. Follow-up MRI is recommended at 6 months and then annually for patients with macroadenomas and every 1–2 years for patients with microadenomas. Periodic clinical and biochemical testing for non-progressive adenomas can be tailored according to the presence of symptoms and the rate of tumor growth. Finally, a study on 34 children with Rathke's cleft cysts reported CPP in 46% of them. The authors concluded that the treatment outcomes were similar between the CPP patients with and without Rathke's cleft cysts (26).

In 2000, De Sanctis et al. (4) reported brain abnormalities in 18.4% of 304 CPP girls, the prevalence of brain abnormalities in different age groups were: 32.3% of girls younger than 4 years, 18.2% of girls 4–6.9 years, 16% of girls 7–7.9 years. However, most patients with brain abnormalities had previous known associations of CPP with intracranial pathology or other coincidental or related clinical findings, such as developmental delay, dysmorphic facial features, intrauterine growth retardation, etc. Chalumeau el al. (27) reported 443 girls with CPP in France, 8% had an occult intracranial lesion, although the prevalence decreasing with age: 26.85% in age below 6 years, 2.17% in age 6–6.9 years, 1.66% in age 7–7.9 years. However, 17% of 35 patients who had occult pathological CNS lesions were older than 6 years at puberty onset, including astrocytoma, hamartoma, and arachnoid cyst. Therefore, the authors developed an algorithm to identify the risk of organic CPP, age below 6 years or estradiol above 45th percentile indicate high risk for organic brain lesion in European population.

Our findings support the decreasing trend of the prevalence of intracranial lesions by age. None of the girls with intracranial lesions requiring intervention besides GnRH agonist therapy. It may be cautious to reconsider the routine use of brain MRI to screen all girls with CPP, especially in those who are healthy without neurologic symptoms.

MRI is a technique to employ and may impose risks on the patients due to the use of sedatives (in children) and intravenous contrast agents. Indeed, the US Food and Drug Administration previously cautioned against the use of gadolinium-based contrast agents in MRI as gadolinium deposits may remain in the brain for years, especially with the usage of linear gadolinium based contrast agents rather than macrocyclic agents. To avoid potential risks, all healthcare professionals should carefully consider the need for MRI (28, 29).

This study had several limitations, the first of which was its retrospective nature. Second, the study enrollees were treated at a single medical center. Third, the prevalence of intracranial lesions among patients who did not undergo brain MRI remains unclear. Finally, the results of this study may have been influenced by other factors such as race and differences in medical and health insurance systems.

## Data Availability Statement

The datasets generated for this study are available on request to the corresponding author.

## Author Contributions

C-FC collected and interpreted the data. C-JW reviewed and interpreted the MRI and CT images. Y-PC dealt with data mining and statistic and revised the manuscript. F-SL designed and supervised the study and interpreted the results. All authors wrote and approved the manuscript.

## Conflict of Interest

The authors declare that the research was conducted in the absence of any commercial or financial relationships that could be construed as a potential conflict of interest.
