# Supplementary material for: Pathological and Incidental Findings in 403 Taiwanese Girls With Central Precocious Puberty at Initial Diagnosis
Source: Front Endocrinol (Lausanne). 2020 May 5;11:256. doi: 10.3389/fendo.2020.00256 (PMC7214687; doi:10.3389/fendo.2020.00256)
Supplement: Supplementary file 1 [file Table_1.docx]

Supplementary Appendix

**Table 1**. Descriptive characteristics of 403 girls with newly diagnosed central precocious puberty

|  | **Mean ± SD** | **Range** |
| --- | --- | --- |
| **Age at diagnosis(years)** | 6.51±1.50 | 0.63-8.0 |
| **Bone age at diagnosis(years)** | 7.96±2.21 | 0.5-12.6 |
| **BA-CA(years)** | 1.43±1.41 | -1.97-6.17 |
| **BMI(kg/m2)** | 17.20±2.06 | 12.78-23.44 |
| **Height SDS** | 0.78±1.06 | -1.99-4.34 |
| **Hight SDS-MPH SDS** | 1.05±0.99 | -2.16-4.42 |
| **Uterus size(cm)** | 3.71±1.13 | 1.5-8.2 |
| **Right ovary size(cm)** | 2.0±0.62 | 0.45-4.1 |
| **Left ovary size(cm)** | 1.9±0.58 | 0-4.2 |
| **Basal LH(mIU/mL)** | 1.76±2.03 | 0.1-14.3 |
| **Basal FSH(mIU/mL)** | 4.16±3.0 | 0.5-38.8 |
| **Estradiol(mIU/mL)** | 22.31±26.19 | 0.76-315.4 |
| **Peak LH(mIU/mL)** | 21.69±29.57 | 5-518.5 |
| **Peak FSH(mIU/mL)** | 17.35±7.07 | 0.4-63 |
| **LH/FSH peak ratio** | 1.51±1.48 | 0.18-17 |
